# Re-validation and re-description of *Tasakoreana* (Wesołowska, 1981) (Araneae, Salticidae)

**DOI:** 10.3897/BDJ.10.e87443

**Published:** 2022-07-07

**Authors:** Chi Jin, Siyuan Liu, Lixin Wang, Manping Luo, Kai Chen

**Affiliations:** 1 School of Landscape and Ecological Engineering, Hebei University of Engineering, Handan, China School of Landscape and Ecological Engineering, Hebei University of Engineering Handan China

**Keywords:** Spider, East Asia, synonymy, taxonomy, China

## Abstract

**Background:**

*Tasa* Wesołowska, 1981 is a small chrysilline genus distributed in East Asia, with two currently known species: *T.davidi* (Schenkel, 1963) and *T.nipponica* Bohdanowicz & Prószyński, 1987, both species having been recorded in China.

**New information:**

The combination *Tasakoreana* (Wesołowska, 1981) comb. rev. is re-validated, based on the characteristics of the copulatory organs of both sexes. A re-description and diagnostic photographs are also provided.

## Introduction

The jumping spider species *Tasakoreana* (Wesołowska, 1981) has a complicated taxonomic history and even now, its female is still misplaced in another genus, *Nepalicius* Prószyński, 2016, under the name *N.koreanus* (Wesolowska, 1981).

Initially, this species was established by Wesołowska (1981) on the basis of a female specimen collected from North Korea and named as *Pseudiciuskoreanus* Wesołowska, 1981. Then, its female was reported in Japan and the species was transferred to the genus *Icius* Simon, 1876 by Yaginuma (1986). After that, this species was reported twice more under the name *Iciuskoreanus* (Wesolowska, 1981) and both reports described its newly-discovered male: Bohdanowicz and Prószyński (1987) provided a detailed drawing of the vulva, based on the Japanese female and the described male was later identified as *Pseudiciustokarensis* (Bohdanowicz & Prószyński, 1987) by Suguro and Yahata (2014); the male, described as *Iciuskoreanus* in Xiao (1993) from China, was also affirmed by Suguro and Yahata (2014) to be misidentified and considered to belong to a different species. After Xiao (1993), this species has been reported four times in China under the name *Pseudiciuskoreanus*, both females and males being reported at the same time, but without exception, the males in these reports all followed Xiao (1993) and were misidentified (Peng et al. 1993, Song et al. 1999, Yin et al. 2012, Peng 2020). Ono et al. (2009) also reported *Pseudiciuskoreanus*, based on both female and male specimens, but the male was misidentified and belonged *P.tokarensis*. Suguro and Yahata (2014) matched the female *Pseudiciuskoreanus* with the male *Tasanipponica* Bohdanowicz & Prószyński, 1987 and established a new combination *Tasakoreana* (Wesołowska, 1981), based on sequencing data of the mitochondrial COI gene and somatic patterns. In the specimens they examined, multiple pairs of males and females were collected at the same time, which undoubtedly provided evidence for this combination. Prószyński (2016) rejected the above combination, re-instated the male of *Tasakoreana* to *T.nipponica* and removed the female into a newly-established genus *Nepalicius* Prószyński, 2016, named as *N.koreanus* (Wesołowska, 1981).

In a field investigation, we collected a pair of jumping spiders and, after identification, we found that the male matches with *Tasanipponica* and the female with *Pseudiciuskoreanus*, which is consistent with Suguro and Yahata (2014). Therefore, we re-validated the taxonomic status of *Tasakoreanus* (Wesołowska, 1981) and re-described this species.

## Materials and methods

All measurements are given in millimetres (mm). Leg measurements are shown as total length (femur + patella + tibia + metatarsus + tarsus). The epigyne was removed and cleared in a pancreatin solution (Álvarez-Padilla and Hormiga 2007). All specimens were preserved in 75% alcohol and were examined and measured with a Leica M205A stereomicroscope. The colour in the description is based on the preserved specimens. Photographs of the habitus were captured using a Leica M205A stereomicroscope, equipped with a DFC550 CCD camera; photographs of the copulatory organs were taken by an Olympus BX53 microscope, equipped with a Kuy Nice CCD Camera and were stacked by the Helicon Focus 7 software. The specimens used in this study are deposited in the School of Landscape and Ecological Engineering, Hebei University of Engineering (HEBEU) in Handan, China.

The following abbreviations are used in the description: AERW—anterior eye row width; ALE—anterior lateral eyes; AME—anterior median eye; EFL—eye field length; PERW—posterior eye row width; PLE—posterior lateral eye; PME—posterior median eye; RTA—retrolateral tibial apophysis. Abbreviations used in the leg spination: d—dorsal; dt—dorsal terminal; pv—proventral; rv—retroventral; vt—ventral terminal.

## Taxon treatments

### Tasa koreanus

(Wesołowska, 1981), comb. rev.

C8E8B0B7-8735-5497-88DF-CDB4A7678859

Pseudicius koreanus Wesołowska, 1981, see Wesołowska (1981): 60, f. 52–55, ♀ (female holotype from North Korea: Pyeongyang, deposited in the Institute of Zoology of the Polish Academy of Science in Warszawa, not examined); Peng et al. (1993): 192, f. 677–679, ♀♂ (only female; male misidentified = *Iciuskoreanus* Xiao, 1993); Song et al. (1999): 542, f. 312M, 313O, 328P, ♀♂ (only female; male misidentified = *Iciuskoreanus* Xiao, 1993); Ono et al. (2009): 570, f. 104–106, ♀♂ (only female; male misidentified = *P.tokaraensis*); Yin et al. (2012): 1451, f. 791a–i, ♀♂ (only female; male misidentified = *Iciuskoreanus* Xiao, 1993); Peng (2020): 367, f. 264a–i, ♀♂ (only female; male misidentified = *Iciuskoreanus* Xiao, 1993).
Icius koreanus (Wesołowska, 1981), see Yaginuma (1986): 233, f. 130.1, ♀ (female transferred from *Pseudicius*); Bohdanowicz and Prószyński (1987): 67, f. 72–73, ♀♂ (only female; male misidentified = *P.tokaraensis*); Xiao (1993): 123, f. 1–6, ♂ (male misidentified = unknown species).
Tasa nipponica Bohdanowicz & Prószyński, 1987, see Bohdanowicz and Prószyński (1987): 143, f. 300–303, ♂ (male holotype from Japan: Kochi Pref., depository cannot be traced, not examined); Chen and Zhang (1991): 318, f. 339, ♂; Seo (1992): 183, f. 13–16, ♂; Ikeda (1995): 163, f. 15–20, ♂; Song et al. (1999): 561, f. 319N–O, ♂; Cho and Kim (2002): 136, f. 89, 198–199, ♂; Namkung (2002): 590, f. 43.34a–b, ♂; Namkung (2003): 594, f. 43.34a–b, ♂; Ono et al. (2009): 574, f. 149–153, ♂; Kim and Lee (2014): 143, f. 102A–C, ♂; Prószyński (2016): 4 (removed from synonym of *Tasakoreana*, rejected here); Prószyński (2017): 31, f. 13L, ♂; Peng (2020): 468, f. 343a–b, ♂.
Pseudicius tokaraensis (Bohdanowicz & Prószyński, 1987) - Suguro and Yahata (2014): 90, f. 10, 16–17, 21–22, ♂ (male misidentified per Prószyński, 2016: 4, rejected here).
Tasa koreana Suguro & Yahata, 2014, see Suguro and Yahata (2014): 94, f. 12, 14, 25, 27–28, ♀♂ (female transferred from *Pseudiciuskoreanus*; male transferred from *Tasanipponica*).

#### Materials

- **Type status:** Other material. **Occurrence:** recordedBy: Chi Ji; individualCount: 1; sex: male; lifeStage: adult; **Taxon:** scientificName: *Tasakoreanus* (Wesołowska, 1981); namePublishedIn: Suguro, T. and Yahata, K. 2014. Acta Arachnologica, 63(2): 94.; class: Arachnida; order: Araneae; family: Salticidae; genus: Tasa; **Location:** continent: Asia; country: China; countryCode: CN; stateProvince: Hebei; county: Hanshan District; municipality: Handan; locality: Fuyang Park; verbatimElevation: 53 m; verbatimLatitude: 36.5927°N; verbatimLongitude: 114.5102°E; **Identification:** identifiedBy: Chi Jin; **Event:** samplingProtocol: by hand; year: 2019; month: 6; day: 1; habitat: tree trunk
- **Type status:** Other material. **Occurrence:** recordedBy: Chi Ji; individualCount: 1; sex: female; lifeStage: adult; **Taxon:** scientificName: *Tasakoreanus* (Wesołowska, 1981); namePublishedIn: Suguro, T. and Yahata, K. 2014. Acta Arachnologica, 63(2): 94.; class: Arachnida; order: Araneae; family: Salticidae; genus: Tasa; **Location:** continent: Asia; country: China; countryCode: CN; stateProvince: Hebei; county: Hanshan District; municipality: Handan; locality: Fuyang Park; verbatimElevation: 53 m; verbatimLatitude: 36.5927°N; verbatimLongitude: 114.5102°E; **Identification:** identifiedBy: Chi Jin; **Event:** samplingProtocol: by hand; year: 2019; month: 6; day: 1; habitat: tree trunk

#### Description

**Figure 1.**
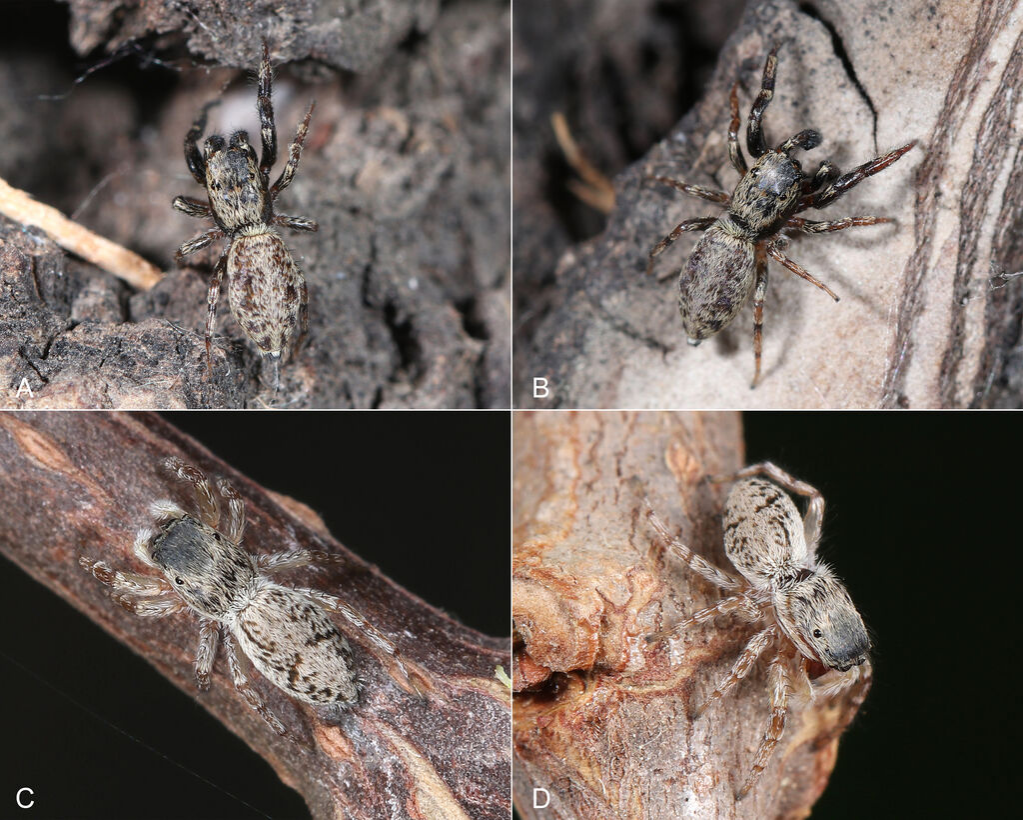
Living spiders of *Tasakoreanus* (Wesołowska, 1981): **A–B** male; **C–D** female. Photographs by Chi Jin.

**Figure 2.**
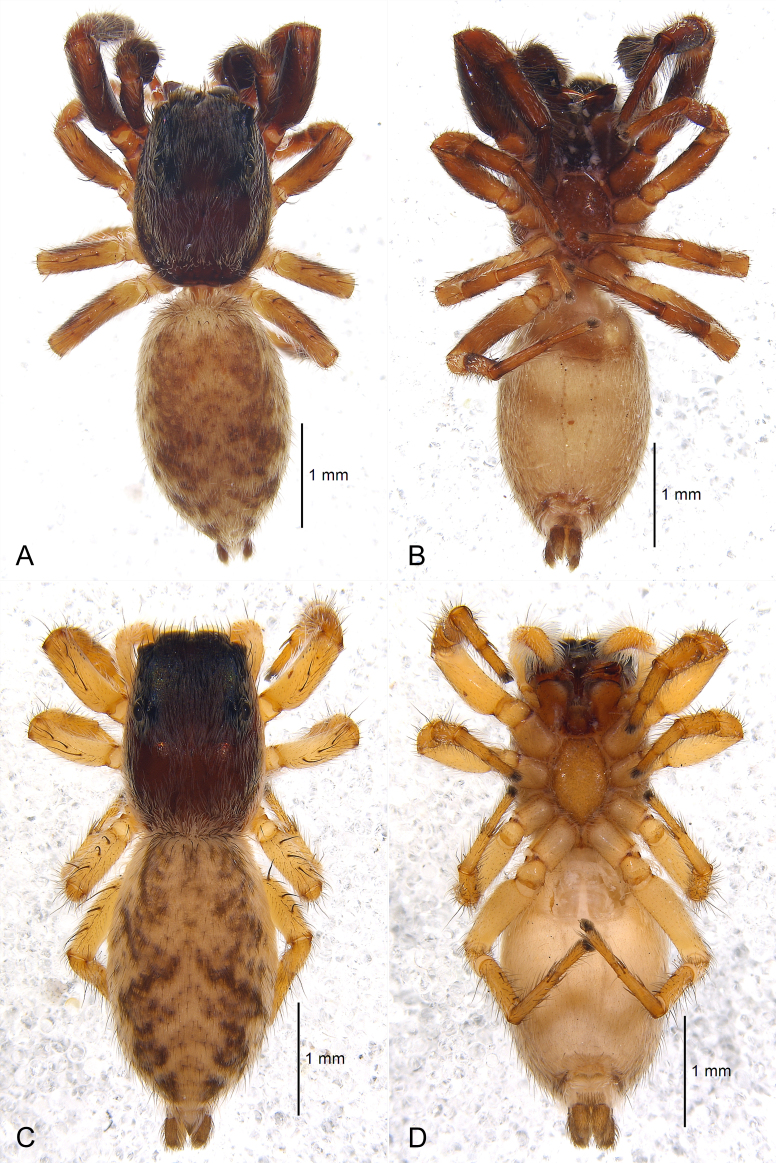
*Tasakoreanus* (Wesołowska, 1981): **A** male habitus, dorsal view; **B** same, ventral view; **C** female habitus, dorsal view; **D** same, ventral view.

**Figure 3.**
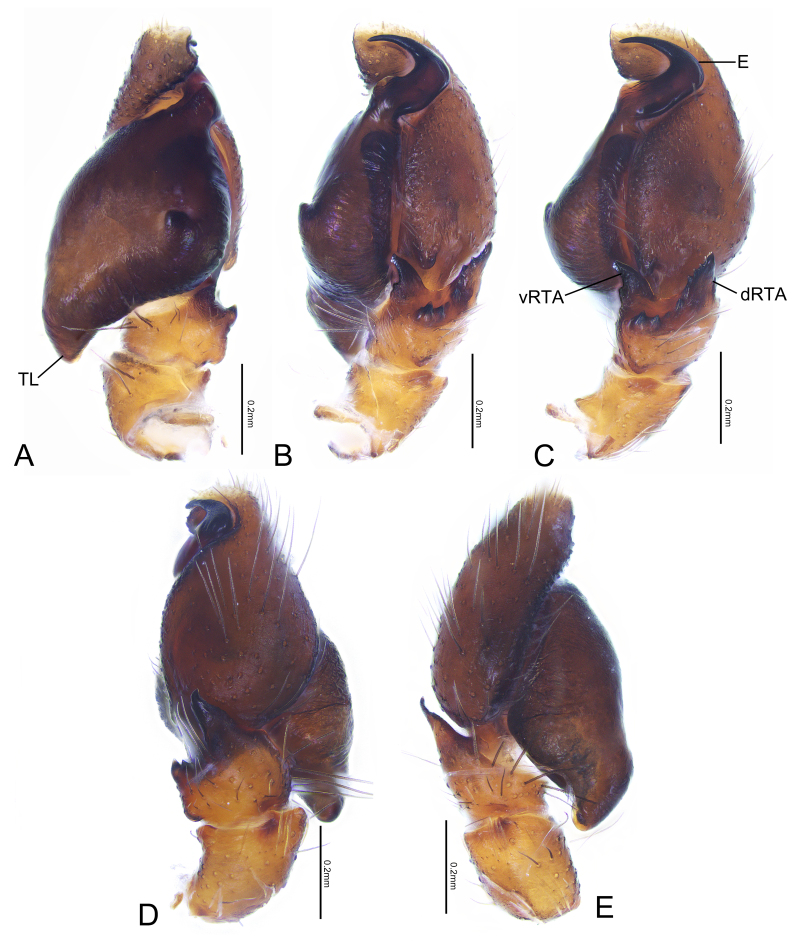
*Tasakoreanus* (Wesołowska, 1981): **A** male left palp, ventral view; **B** same, retrolateral-ventral view; **C** same, retrolateral view; **D** same, dorsal view; **E** same, prolateral view. Abbreviations: E—embolus; dRTA—dorsal branch of retrolateral tibial apophysis; TL—tegular lobe; vRTA—ventral branch of retrolateral tibial apophysis.

**Figure 4.**
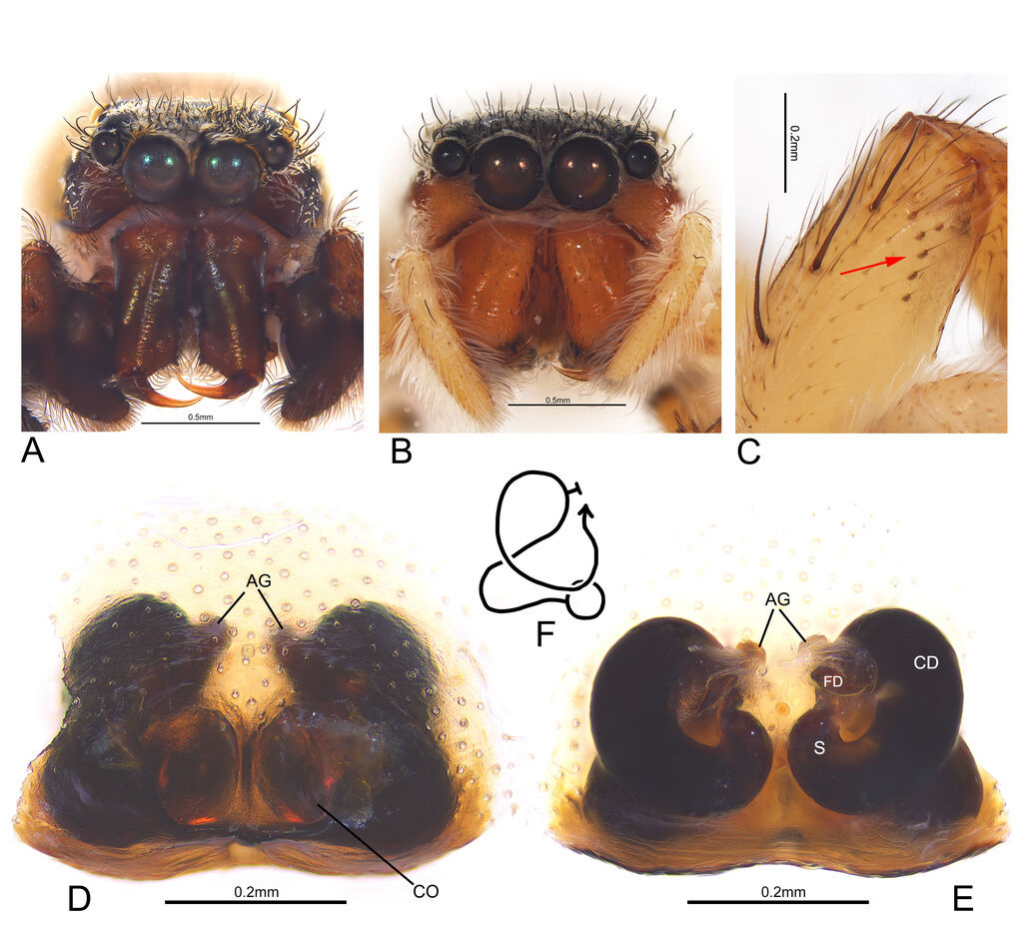
*Tasakoreanus* (Wesołowska, 1981): **A** male cephalothorax, frontal view; **B** female cephalothorax, frontal view; **C** female left femur I, prolateral view, red arrow points to the sclerotic and raised sockets; **D** epigyne, ventral view; **E** vulva, dorsal view; **F** schematic of internal duct system, dorsal view. Abbreviations: AG—accessory gland; CD—copulatory duct; CO—copulatory opening; FD—fertilisation duct; S—spermatheca.

Male (Fig. 1A and B, Fig. 2A and B, Fig. 3A–E and Fig. 4A). Total length 4.33. Carapace 1.89 long, 1.31 wide. Abdomen 2.44 long, 1.44 wide. Eye sizes: AME 0.29, ALE 0.13, PME 0.04, PLE 0.14, AERW 0.99, PERW 1.00, EFL 0.70. Legs: I 3.58 (1.06 + 0.74 + 0.85 + 0.59 + 0.34), II 2.68 (0.82 + 0.52 + 0.54 + 0.47 + 0.33), III 2.62 (0.80 + 0.48 + 0.50 + 0.50 + 0.34), IV 3.37 (1.06 + 0.54 + 0.71 + 0.67 + 0.39).

Carapace dark brown, elongate and flat, nearly rectangular; dorsal surface covered with dense white hairs (Fig. 2A); lateral surface hairless (Fig. 1B). Fovea indistinct, short and longitudinal. Eye field black, surrounded by sparse black setae. Chelicerae dark brown (Fig. 4A), with two promarginal and one retromarginal teeth. Labium black and as wide as long. Endites dark brown. Sternum brown. Abdomen elongated oval, grey-white, covered with dense white hairs and sparse black hairs, with a pair of light brown, mottled stripes running through entire dorsum; dorsal scutum absent (Fig. 2A); venter with two columns of indistinct sclerites between epigastric furrow and spinnerets; spinnerets brown (Fig. 2B). Leg I dark brown; legs II–IV yellowish-brown (Fig. 2B). Palps dark brown. Spination of legs as shown in Table 1.

Palp (Fig. 3A–E). Bulb nearly triangular in ventral view, retrolateral side with a small protrusion; sperm duct extending along margin; tegular lobe curved prolaterally. Embolus stubby and hook-shaped. RTA bifurcated, ventral branch as long as dorsal branch, blunt; dorsal ramus well-developed, pointed with about ten teeth of various sizes, along ventral margin, distributed from RTA base to tip of dorsal branch. Cymbium distally curled retrolaterally, forming a groove to accommodate embolus.

Female (Fig. 1C and D, Fig. 2C and D and Fig. 4B–F). Total length 4.17. Carapace 1.84 long, 1.23 wide. Abdomen 2.33 long, 1.50 wide. Eye sizes: AME 0.28, ALE 0.14, PME 0.04, PLE 0.14, AERW 0.98, PERW 1.06, EFL 0.73. Legs: I 2.70 (0.87 + 0.56 + 0.53 + 0.45 + 0.29), II 2.36 (0.74 + 0.50 + 0.44 + 0.40 + 0.28), III 2.59 (0.81 + 0.46 + 0.46 + 0.51 + 0.35), IV 3.34 (1.03 + 0.54 + 0.69 + 0.64 + 0.44).

Carapace with dark brown thorax, bright brown lateral sides and black eye field; almost entirely covered with dense white hairs, but hairless at junction of dorsal and lateral surface (Fig. 1D). Chelicerae bright brown (Fig. 4B). Labium brown and as wide as long. Endites bright brown. Sternum yellowish-brown. Abdomen grey-white, dorsal stripes similar to males, but darker in colour (Fig. 2C); venter without sclerites; spinnerets yellowish-brown (Fig. 2D). Legs and palps light yellow (Fig. 2D); distal prolateral portion of femur I with 5–6 sclerotised hairs from raised sockets in a Y-shaped arrangement (Fig. 4C). Spination of legs as shown in Table 1. Other somatic characters as in male.

Epigyne (Fig. 4D): copulatory openings located posteriorly, large and oval, sharing a sclerotised posterior margin. Vulva (Fig. 4E and F): copulatory ducts stout, twisted in S-shape; spermathecae small, close to each other and have no obvious boundary with copulatory ducts; accessory glands short, located below anterior margin of copulatory ducts.

#### Diagnosis

This species closely resembles *Tasadavidi* (Schenkel 1963), but can be distinguished from it by: 1) the two branches of RTA nearly equal in length, whereas the ventral branch is much shorter than the dorsal one in *T.davidi* (Fig. 3C, Wesołowska 1981: 157, fig. 90); 2) the accessory glands short and located below the anterior margin of the copulatory ducts, whereas they are longer and located significantly above the anterior margin of the copulatory ducts in *T.davidi* (Fig. 4C and D, Peng et al. 2000: 19, fig. 17).

#### Distribution

China (Hebei, Zhejiang Province), Japan, Korea.

#### Biology

Habitat on tree trunks or branches.

#### Taxon discussion

The female of this species closely resembles the female of *T.davidi*, the type species of the genus *Tasa*, justifying its transfer to *Tasa*. Additionally, the stubby embolus of the male (sub *T.nipponica*) corresponds with the large copulatory openings and stout copulatory ducts of its female (sub *P.koreanus*) (Figs 3, 4). Relatively, in *N.koreanus*, the slender embolus of the male (sub *P.tokaraensis*) clearly does not correspond with the large copulatory openings and stout copulatory ducts of its so-called female (sub *P.koreanus*) (Prószyński 2016: 22, figs. 7C–D). The figures of the epigyne and vulva of this species, presented in Suguro and Yahata (2014) (95, figs. 29–30), are more similar to *T.davidi* than to *T.koreanus*, possibly due to different illustration or processing methods, which will require future examination of their specimens to confirm.

## Supplementary Material

XML Treatment for Tasa koreanus

## Figures and Tables

**Table 1.** Spination of legs of *Tasakoreanus* (Wesołowska, 1981).

	Leg	Femur	Tibia	Metatarsus
♂	Ⅰ	d 3	–	pv 2 rv 2
	Ⅱ	d 3	–	pv 1 rv 2
	Ⅲ	d 3	pv 1 rv 1	dt 2 vt 3
	Ⅳ	d 3	pv 1 rv 1	dt 2 vt 3
♀	Ⅰ	d 3	pv 1	pv 2 rv 2
	Ⅱ	d 3	–	pv 1 rv 2
	Ⅲ	d 3	pv 1 rv 1	dt 2 vt 3
	Ⅳ	d 3	pv 2 rv 1	pv1 d1 dt 2 vt 3
